# Effects of Accelerated Aging on Thermal, Mechanical and Shape Memory Properties of Cyanate-Based Shape Memory Polymer: III Vacuum Thermal Cycling

**DOI:** 10.3390/polym15081893

**Published:** 2023-04-14

**Authors:** Zhongxin Ping, Fang Xie, Xiaobo Gong, Liwu Liu, Jinsong Leng, Yanju Liu

**Affiliations:** 1 Center for Composite Materials and Structures, Harbin Institute of Technology, Harbin 150080, China; 2 Department of Materials Science and Engineering, Harbin Institute of Technology at Weihai, Weihai 264209, China; 3 School of Naval Architecture and Ocean Engineering, Harbin Institute of Technology at Weihai, Weihai 264209, China; 4 Department of Astronautical Science and Mechanics, Harbin Institute of Technology, Harbin 150001, China

**Keywords:** cyanate-based SMP, vacuum thermal cycling, shape memory effect, thermal properties, mechanical properties, aging resistance

## Abstract

Shape memory polymers (SMPs) with intelligent deformability have shown great potential in the field of aerospace, and the research on their adaptability to space environments has far-reaching significance. Chemically cross-linked cyanate-based SMPs (SMCR) with excellent resistance to vacuum thermal cycling were obtained by adding polyethylene glycol (PEG) with linear polymer chains to the cyanate cross-linked network. The low reactivity of PEG overcame the shortcomings of high brittleness and poor deformability while endowing cyanate resin with excellent shape memory properties. The SMCR with a glass transition temperature of 205.8 °C exhibited good stability after vacuum thermal cycling. The SMCR maintained a stable morphology and chemical composition after repeated high–low temperature cycle treatments. The SMCR matrix was purified by vacuum thermal cycling, which resulted in an increase in its initial thermal decomposition temperature by 10–17 °C. The continuous vacuum high and low temperature relaxation of the vacuum thermal cycling increased the cross-linking degree of the SMCR, which improved the mechanical properties and thermodynamic properties of SMCR: the tensile strength of SMCR was increased by about 14.5%, the average elastic modulus was greater than 1.83 GPa, and the glass transition temperature increased by 5–10 °C. Furthermore, the shape memory properties of SMCR after vacuum thermal cycling treatment were well maintained due to the stable triazine ring formed by the cross-linking of cyanate resin. This revealed that our developed SMCR had good resistance to vacuum thermal cycling and thus may be a good candidate for aerospace engineering.

## 1. Introduction

Stimuli-responsive SMPs can recover from programmed temporary shapes to their original shapes after being subjected to external stimuli such as light, heat, magnetism, and electricity. They exhibit recoverable deformation capabilities, enhanced mechanical properties, and controllable remote actuation ability. Based on this, SMPs have broad application prospects in aerospace engineering [1,2], biomedical engineering [3,4], flexible electronics [5,6], soft robotics [7,8], 4D printing [9,10], and deformable device design [11,12]. In aerospace, deployable space structure technology was the main means to solve the contradiction between the structure of large-scale spacecrafts and the limitation of the rocket launch envelope [13]. Intelligent modern aerospace technology also presents unique requirements for SMPs. For example, in order to enhance the traditional aircraft, which can only perform a single flight mission, a deformable, folding aircraft with an intelligent, adaptive wing based on shape memory alloy was developed [14]. However, shape memory alloys have disadvantages, such as a low deformation rate and high density. Therefore, SMPs with low density and high deformation rates have become a rigid demand in the aerospace field. Studies on the application of SMPs in space detectors with lightweight, low-cost, and controllable deformation have deepened. The SMP-based space deployable structure can be preprogrammed into a temporary shape with a smaller volume, and it can then be easily restored to the original shape through external stimulation after entering the target orbit. However, the harsh space environment can cause irreversible damage to spacecraft materials and structures [15]. The space environment refers to the natural and induced environment of Earth space and interplanetary space, including blackness, low temperature, vacuum, solar electromagnetic radiation, charged particle radiation, neutral atmosphere, magnetic field, ionosphere, plasma, space debris and micrometeoroids, and induced environmental pollution [16]. Today, additive manufacturing technologies, such as 4D printing, make on-demand sample manufacturing possible in the aerospace field due to their inherent high degree of freedom. However, the unknown spatial stability of SMP substrates limits their development [17,18,19,20] and necessitates that SMPs used in the construction of spacecraft must undergo strict space environment reliability tests before practical application.

Most of the reported SMPs had excellent shape memory effects. However, research on their adaptability to the space environment is so far poor, which greatly limits the application of SMPs in the aerospace field. The harsh space environment—such as vacuum, low temperature, solar ultraviolet (UV) radiation, charged particle radiation, atomic oxygen (AO), and geomagnetic field—is an important factor that hinders the long-term application of SMPs in aerospace engineering [21,22,23]. A series of adverse physical and chemical changes occurs in a vacuum thermal cycle environment, which greatly shortens the service life of materials [24].

Cyanate-based SMPs with high glass transition temperature and extremely low dielectric loss have emerged as key aerospace candidates [25,26,27]. Cyanate-based SMPs are widely used in space-deployable structures, such as flexible solar array systems [28]. In our previous study, a series of cyanate-based SMPs prepared using bisphenol A cyanate and two different modifiers (polyethylene glycol and polybutadiene-acrylonitrile) exhibited tunable T_g_ (up to 255 °C) and good shape memory properties [27]. The investigation revealed that the thermomechanical properties and shape memory properties of the prepared cyanate-based SMPs underwent few significant changes during UV irradiation or atomic oxygen irradiation [15,29]. These cyanate-based SMPs had excellent resistance to ultraviolet radiation and anti-atomic oxygen radiation. However, the materials were inevitably subjected to continuous thermal cycling environments within the space environment [30,31], which were particularly damaging. Therefore, in this follow-up study, it was crucial to identify the performance changes in cyanate-based SMPs under continuous vacuum thermal cycling, to support their development for practical applications in the aerospace field.

Chemically cross-linked cyanate-based SMPs were prepared by adding polyethylene glycol with linear polymer chains to the cyanate cross-linked network. The effects of vacuum high- and low-temperature cycles on the properties of cyanate-based SMPs were revealed through the surface morphology, chemical composition, thermal stability, and mechanical properties. The prepared SMCR maintained a stable morphology and chemical composition after repeated vacuum thermal cycling treatments. The initial thermal decomposition temperature of SMCR after vacuum thermal cycling increased by 10–17 °C. The tensile strength of the treated SMCR increased by about 14.5%, the average elastic modulus was greater than 1.83 GPa, and the glass transition temperature increased by 5–10 °C. Furthermore, the average shape fixation rate and average shape recovery rate of the SMCR remained stable after vacuum thermal cycling. In our previous work [15,29], vacuum ultraviolet radiation caused slight changes in the molecular structure of our developed SMCR, while atomic oxygen caused erosion of SMCR only on the surface layer. However, in this study, vacuum UV irradiation and atomic oxygen had little effect on the shape memory and mechanical properties of our developed SMCR. Our developed SMCR had good resistance to ultraviolet radiation, atomic oxygen, and vacuum thermal cycling, and we propose that it represents a good candidate for space-deployable structures.

## 2. Experiment

### 2.1. Materials and Methods

Bisphenol A cyanate monomer was purchased from Jiangdu Wuqiao Resin Factory, China. Polyethylene glycol (PEG, M_n_ = 20,000) was supplied by Tianjin Guangfu Fine Chemical Research Institute, China. The mass ratio of 7.14: 2.86 of cyanate monomer and PEG was completely melted at 100 °C and then stirred mechanically for 20 min to obtain a homogeneous mixture. The mixture was degassed in a vacuum oven for 15 min. The above mixture was injected into a preheated glass mold. The mixture was cured in three steps: holding at 120 °C for 2 h, 180 °C for 2 h, and 210 °C for 5 h. Finally, cyanate-based SMP sheets with a thickness of 2 mm were obtained.

### 2.2. Vacuum Thermal Cycling Test

Vacuum thermal cycling tests were performed using the BZ11 vacuum high- and low-temperature environment experiment system. The exposure was conducted at the vacuum level of 5 × 10^−4^ Pa. For each cycle, SMCR samples were warmed up to 170 °C and held under a vacuum for 1 h, and then cooled down to −170 °C and held for 3 h. A batch of samples was taken out when the number of cycles reached 10, 30, and 50, respectively. The batch of samples was then sealed in vacuum bags for subsequent testing.

### 2.3. Characterization Method

Fourier transform infrared spectroscopy (FTIR) was investigated by using a PECTRUM ONE spectrometer (Perkin Elmer Corporation, Waltham, MA, USA) in the scanning range of 4000–650 cm^−1^. The resolution was 4 cm^−1^, and the scanning times were 8. The test samples were prepared by the KBr disk method.

Thermogravimetric curves (N_2_, at a ramp rate of 10 °C/min, temperature range: 25–800 °C) were recorded using a TGA (Thermal Gravimetric Analyzer)/DSC (Differential Scanning Calorimetry) 1 analyzer (Mettler-Toledo, Greifensee, Switzerland). The test samples (8–10 mg) were obtained from powder scraped off the bare surface of the samples.

Dynamic mechanical analysis (DMA; Q800, TA Instruments, Brooklyn, NY, USA) was used to study the energy storage modulus and tan δ of the samples. Tests were performed in tensile mode heated from 25 °C to 350 °C at a rate of 3 °C/min (amplitude of 10 μm, frequency of 1 Hz, and minimum dynamic force of 0.1 N). Samples (20 × 3 × 1 mm^3^) were shaped by a high-speed engraving machine.

Quasi-static tensile tests were performed on a Zwick / Roell Z010 tensile testing machine (Zwick GmbH & Co. KG, Ulm, Germany) (ambient temperature 25 °C, beam movement speed 5 mm/min). Based on the ASTMD638 standard, the tensile test pieces (type IV) were prepared using a high-speed precision engraving machine. For each sample, tensile testing was performed five times to avoid chance.

The shape memory properties of SMCR before and after thermal cycling treatment were investigated using DMA (DMAQ800, TA Instruments, Brooklyn, NY, USA). For the Nth cycle, the original displacement of the SMCR was defined as D_n0_. The sample was first bent at 210 °C, and the offset was recorded as D_n1_. Then, when the sample was completely cooled to room temperature, the external force was removed, and the displacement value was recorded as D_n2_. The temperature was again increased to 210 °C to obtain the recovered shape, and the displacement value was recorded as *D*_(*n*+1)0_, the initial displacement for the (N + 1)th cycle. The shape fixation rate *R_nf_* and shape recovery rate *R_nr_* for the Nth cycle can be expressed as:

(1) $$R_{nf}=\frac{D_{n2}-D_{n0}}{D_{n1}-D_{n0}}\times 100\%$$

(2) $$R_{nr}=\frac{D_{n2}-D_{\left(n+1\right)0}}{D_{n2}-D_{n0}}\times 100\%$$

## 3. Results and Discussion

### 3.1. Effects of Vacuum Thermal Cycling on Morphology and Chemical Composition

**Figure 1:**
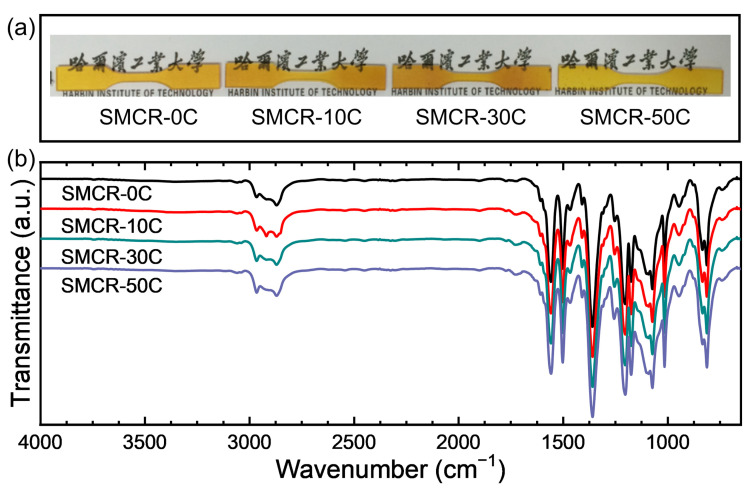
(**a**) Photographs of SMCR without vacuum thermal cycling (SMCR-0C) and after 10 (SMCR-10C), 30 (SMCR-30C), and 50 (SMCR-50C) vacuum thermal cycles; (**b**) FTIR spectra of SMCR before and after vacuum thermal cycling.

The abundant triazine ring structure in the cured cyanate resin led to extremely low mobility of the molecular segment of polymer matrix. Therefore, the shape programming process of cyanate resins was difficult to achieve even above the glass transition temperature. The linear long-chain PEG with low reactive activity was used as a modifier to improve the mobility of a molecular chain segment to achieve a good shape memory performance of SMCR. The strong stretching vibration peak in the range of 1000–1200 cm^−1^ was the carbon–oxygen stretching vibration region of the alcohol hydroxyl group, which indicated that the PEG in the system was largely not involved in the chemical reaction. This proved that the main function of PEG was to reduce the chance of reaction between cyanate monomers to reduce the cross-link density, and thus to impart satisfactory shape memory effect (Figure 1b). The effect of vacuum thermal cycling on the surface morphology of SMCR was surveyed through photographs. As shown in Figure 1a, after 10, 30, and 50 cycles of vacuum thermal cycling at ±170 °C, the color of the SMCR sample had no significant changes, indicating that vacuum thermal cycling did not affect the appearance of the SMCR matrix. Infrared spectroscopy was used to analyze the changes in specific chemical functional groups in cyanate-based SMCR without vacuum thermal cycling and after different times of vacuum thermal cycling. As shown in Figure 1b, the FTIR spectra of the cyanate-based SMCR samples after undergoing vacuum thermal cycling (±170 °C) 10, 30, and 50 times basically overlapped with those of the original samples. The two peaks at 1510–1560 cm^−1^ and 1356 cm^−1^ corresponded to the two triazine rings from the cyanate ester monomer and the curing reaction [30,31]. There was no change in the intensity and peak position of the triazine ring after undergoing continuous vacuum thermal cycling, which was attributed to the extremely high bond energy of the triazine ring. The triazine ring had a six-membered ring structure composed of alternating carbon–nitrogen single bond and carbon–nitrogen double bond, and its high stability was similar to that of a benzene ring. Moreover, no new characteristic peak was generated. These results indicated that vacuum thermal cycling had no significant effect on the chemical composition of cyanate-based SMCR.

### 3.2. Effects of Vacuum Thermal Cycling on Thermal Stability

**Figure 2:**
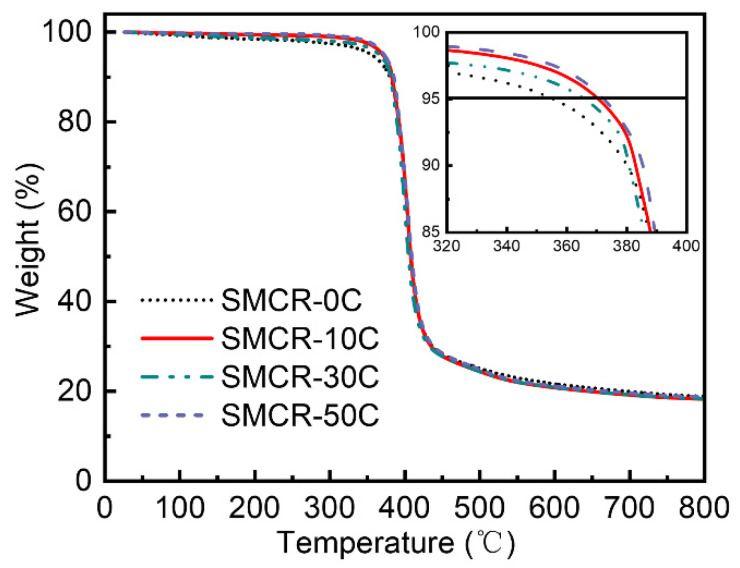
Thermal gravimetric (TGA) curves of SMCR before and after vacuum thermal cycling.

The highest temperature that the polymer could withstand in a determined service environment was used to evaluate the thermal stability of the polymer matrix. The TGA curves of cyanate-based SMCR before and after vacuum thermal cycling are shown in Figure 2. Note that the initial thermal decomposition temperature was defined here as the temperature value when the material mass loss reached 5%, or the sample mass was 95% of the original sample, which reflected the temperature at which the SMP matrix began to thermally decompose. The initial thermal decomposition temperatures of SMCR-0C, SMCR-10C, SMCR-30C, and SMCR-50C were 355.5 °C, 370.4 °C, 365.9 °C, and 372.4 °C, respectively.

In addition to the low initial decomposition temperature of the original sample, the initial decomposition temperature of the other three samples was about 370 °C, which indicated that the thermal stability of the samples actually increased after the vacuum thermal cycling. This can be attributed to the existence of small organic molecules, water, carbon dioxide, or other impurities in the original sample SMCR-0C that had not been treated with vacuum thermal cycling. These small molecules mainly came from raw materials that did not fully participate in the cross-linking reaction and air that adsorbed during sample storage. When undergoing vacuum thermal cycling, these small molecules escaped due to negative pressure, making the sample composition more uniform. That is to say, the vacuum thermal cycling realized the further purification of the cyanate-based SMCR, and the high-purity cyanate-based SMCR formed as a thermosetting resin with a highly chemically cross-linked structure, which had better heat resistance. Small molecules or impurities, water molecules, carbon dioxide, and other less stable substances contained in the sample without vacuum thermal cycling were continuously released in the thermogravimetric experiment, which lowered the initial thermal decomposition temperature of the SMCR-0C. Additionally, when the temperature was higher than 400 °C, the thermogravimetric curves of the samples before and after the vacuum thermal cycling basically overlapped. The carbon residue rate of the four samples at 800 °C was 19%, which showed that there was no difference in the heat resistance of SMCR above 400 °C before and after the vacuum thermal cycling. The vacuum high-temperature environment was helpful for the purification of cyanate-based SMCR and indirectly improved the thermal stability of the matrix.

### 3.3. Effects of Vacuum Thermal Cycling on Mechanical Properties

**Figure 3:**
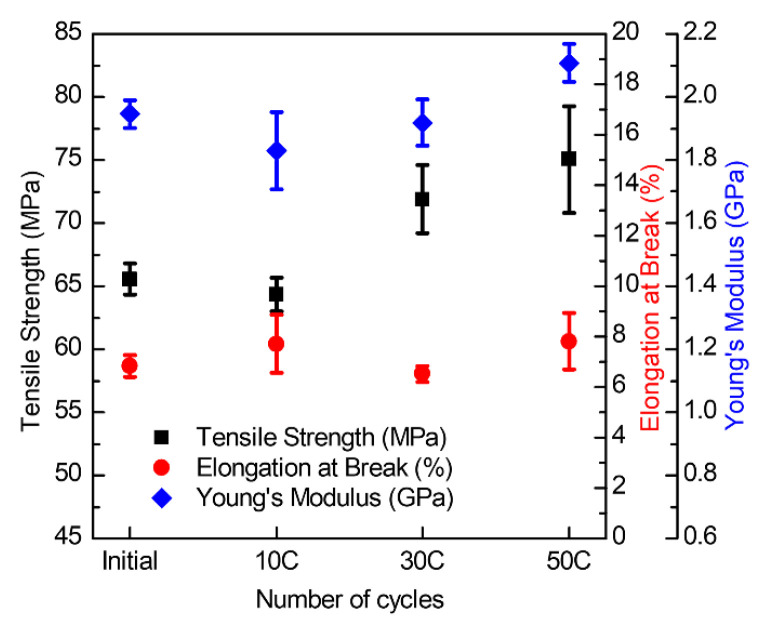
Mechanical properties of SMCR before and after vacuum thermal cycling.

To investigate the effect of vacuum thermal cycling on the mechanical properties of cyanate-based SMCR, static tensile tests were carried out on the samples undergoing different times of vacuum thermal cycling. The mechanical properties of SMCR were compared by measuring tensile strength, elastic modulus, and fracture strain before and after the vacuum thermal cycling (Figure 3). The tensile strength of the SMCR exhibited an overall upward trend before and after being subjected to high- and low-temperature cycles at −170–170 °C in a vacuum environment. The tensile strength of SMCR before vacuum thermal cycling was 65.5 MPa, and after 50 high- and low-temperature cycles, its tensile strength increased to 75.0 MPa, an increase of about 14.5%. Its average breaking strain remained between 6.5 and 7.98%. The tensile modulus of the SMCR decreased slightly after 10 high- and low-temperature cycles, but increased after 30 and 50 cycles. The average elastic modulus of the SMCR was above 1.83 GPa. During the experiment, when the temperature reached a high temperature of 170 °C, it would be kept warm for up to 1 h, and when it reached a low temperature of −170 °C, it would be kept warm for 3 h. The SMCR would undergo a cross-linking reaction at around 170 °C [32,33,34]. Therefore, after the SMCR underwent dozens of vacuum high-temperature relaxation and low-temperature treatments, not only were the impurities in it reduced, but the degree of cross-linking also increased. The molecular structure of the SMCR became denser, which increased the tensile strength and elastic modulus of the SMCR.

### 3.4. Effects of Vacuum Thermal Cycling on Glass Transition Temperature

**Figure 4:**
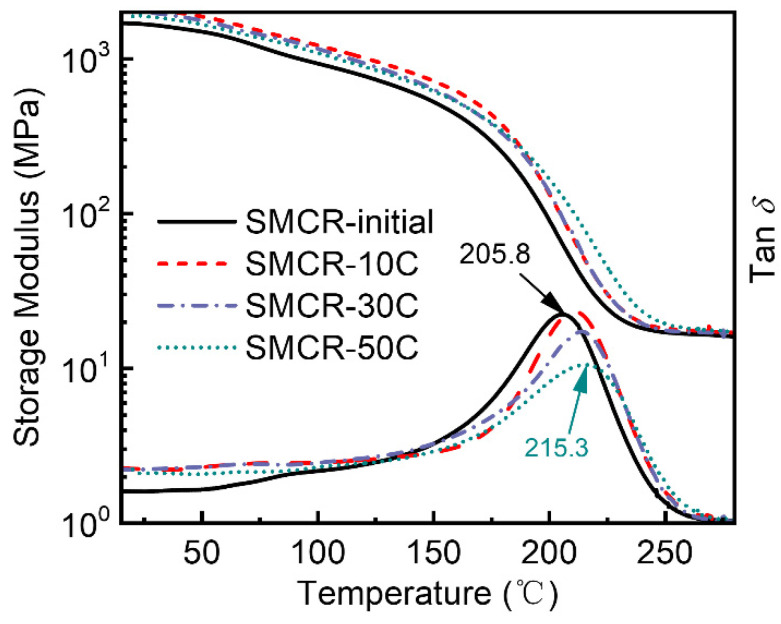
DMA curves of SMCR before and after vacuum thermal cycling.

The glass transition temperature (T_g_) was an important parameter affecting the shape programming of SMPs, and it was necessary to clarify the T_g_ of SMPs during the vacuum thermal cycling process. The tan δ and storage modulus of the SMCR after different times of vacuum thermal cycling (±170 °C) are shown in Figure 4. The temperature corresponding to the peak modulus loss factor of the SMCR was the T_g_. The T_g_ of SMCR treated with vacuum thermal cycling increased by 5–10 °C. Both the storage modulus of the SMCR in the glass state and the storage modulus in the rubber state increased significantly with the increase in the times of vacuum thermal cycling. When SMCR suffered 50 high- and low-temperature cycles, the peak of its tan δ curve moved to the higher temperature, from 205.8 to 215.3 °C. The storage modulus in the high-temperature section was often used as the basis for evaluating the cross-linking density of polymers [35,36,37]. The higher the storage modulus of the polymer in the high-temperature range, the higher the cross-link density. The DMA curve once again proved that the cross-link density of the SMCR increased significantly after undergoing vacuum thermal cycling. The increase in the degree of cross-linking reduced the segment mobility of the SMCR molecule at the same temperature. In other words, a higher temperature was required to obtain the same segmental mobility, or the T_g_ was increased. This was consistent with the previous research.

The switching temperature of the SMCR shape memory process was determined to be ~210 °C (T_trans_ = T_g_), which was considered to be effective for the shape memory process. When the temperature was lower than T_trans_, SMCR was in the glassy state and had a high storage modulus. At this time, the activity of the internal molecular segment of SMCR was low, and its macroscopic shape was locked. When the temperature was higher than T_trans_, the storage modulus of SMCR decreased sharply and tended to be stable with the further increase in temperature. The activity of the internal molecular chain of SMCR was high, which was reflected in the programmability of its macroscopic shape. At room temperature, SMCR can be regarded as a typical hard plastic. At this time, the molecular chain of SMCR was in the lowest energy state. When the temperature was greater than T_trans_, SMCR transformed into a soft elastomer, which led to easy deformation under external force. This macroscopic deformation corresponded to a conformational change in the molecular chain, resulting in an enhanced energy state of the SMCR. When the temperature was less than T_trans_, the deformation of SMCR was maintained even when the external force was removed. It can be simply understood that the molecular chain of the deformed SMCR froze after cooling down, the SMCR matrix maintained a dynamic barrier in a high-energy state, and the energy was stored. Once this kinetic barrier was removed due to increased segment mobility, the stored energy was released, driving the molecular chain back to a lower energy state. This was manifested in the macroscopic shape of the SMCR returning to its original shape. In brief, the driving force of the shape memory behavior of SMCR came from rubber elasticity, that is, the elastic resilience stored in the polymer chain when the polymer network structure was programmed to a temporary shape.

### 3.5. Effects of Vacuum Thermal Cycling on Shape Memory Effect

**Figure 5:**
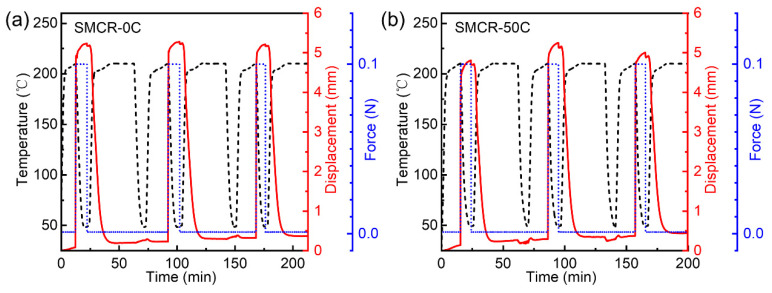
The effect of vacuum thermal cycling on shape memory effect: (**a**) SMCR-0C, (**b**) SMCR-50C.

Space-deployable structures based on SMPs have long been irradiated before reaching their target orbit [38,39], which means that the shape memory effect of SMPs may have changed. The DMA-force control-three-point bending mode was used to monitor the temperature, force, and midpoint displacement of SMCR during shape recovery. As shown in Figure 5, the shape memory effect was carried out on SMCR-0C (a) without vacuum thermal cycling and (b) subjected to 50 vacuum thermal cycles. For each shape memory cycle, the SMCR was first heated to 210 °C, and a force of 0.1 N was applied to the midpoint to obtain a certain displacement. Then, the temperature was lowered to fix the shape, and the external force was removed. Finally, the sample was heated to 210 °C again, and it recovered its original shape without external force.

**Figure 6:**
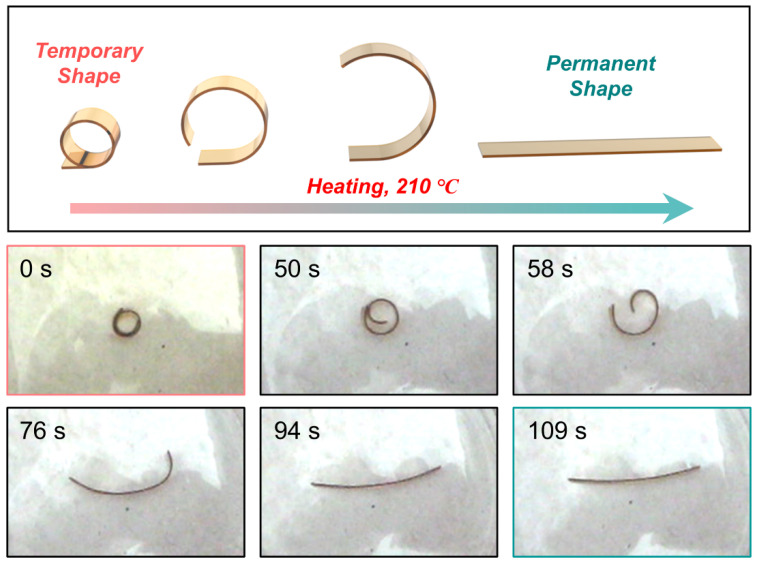
Shape recovery process for SMCR-50 C with toroidal temporary shape.

In agreement with previous studies [40,41,42,43,44], the shape fixation rates of the three shape memory cycles of the SMCR-0C were 97.8%, 97.9%, and 97.1%, with an average shape fixation rate of 97.6%. Their shape recovery rates were 96.9%, 97.0%, and 99.0%, with an average shape recovery rate of 97.6%. After 50 vacuum thermal cycles, the shape fixation rates of the three shape memory cycles of the SMCR-50C were 97.7%, 97.8%, and 97.8%, with an average shape fixation rate of 97.8%; the shape recovery rates were 95.5%, 99.0%, and 98.6%, with an average shape recovery rate of 97.7% (Table 1). The recovery process of cyanate SMCR-50C in a high-temperature furnace is shown in Figure 6. When putting the SMCR-50C fixed in the “ring” shape into a heating furnace at 210 °C, we observed that the SMCR-50C returned to a linear strip within 109 s, and its shape recovery rate reached 100%. These results showed that there was no obvious change in the shape memory fixation rate and recovery rate of SMCR after 50 vacuum thermal cycles, and the repeatability was good, indicating that the vacuum thermal environment had no adverse effect on the shape memory effect of the SMCR.

According to our previous work, vacuum ultraviolet radiation resulted in slight changes in the molecular structure of our developed SMCR, yet the tensile strength and elastic modulus remained essentially unchanged at 66 ± 2 MPa and 1940 ± 80 MPa, respectively. In the literature, the average shape fixation rate and the average shape recovery rate after ultraviolet irradiation were above 97.6% [15]. Furthermore, atomic oxygen only eroded the surface layer of SMCR, and its tensile strength and elastic modulus remained almost unchanged at 66 MPa and 2000 MPa, respectively. The shape memory properties remained excellent after atomic oxygen irradiation, and the average shape fixation and shape recovery rates were 98.2% and 99.3%, respectively [29]. The results from our previous work, combined with the present work, showed that our developed SMCR had good resistance to ultraviolet radiation, atomic oxygen, and vacuum thermal cycling.

## 4. Conclusions

In this work, chemically cross-linked cyanate-based SMPs were prepared by adding PEG with linear polymer chains to the cyanate cross-linked network. The cyanate-based SMCR with a stable triazine ring cross-linked structure maintained excellent mechanical properties, thermal stability, and shape memory properties after continuous vacuum thermal cycling. The prepared SMCR maintained a stable morphology and chemical composition after repeated thermal cycle treatments. The initial thermal decomposition temperature of SMCR increased by 10–17 °C after vacuum thermal cycling treatment. The tensile strength of the treated SMCR increased by about 14.5%, the average elastic modulus was greater than 1.83 GPa, and the glass transition temperature increased by 5–10 °C. The vacuum thermal cycling process increased the cross-link density of SMCR molecules and improved the performance of the polymer matrix. Furthermore, vacuum UV irradiation and atomic oxygen only slightly affected the molecular structure and surface morphology of SMCR, and hardly changed its shape memory and mechanical properties [15,29]. This revealed that our developed SMCR had good adaptability to the space environment and represents a good candidate for space-deployable structures.

## Figures and Tables

**Table 1:** Input and output parameters of the shape memory cycle.

Input Parameter		Output Parameter	SMCR-0C	SMCR-50C
Programming Temperature (°C)	~210			
Loading Mode	DMA-force control-three-point bending mode	Average Shape Fixation Rate (%)	97.6	97.8
Deformation Rate	Midpoint displacement 5 mm			
Heating Rate (°C/min)	~7.36			
Load Holding Time (min)	~10	Average Shape Recovery Rate (%)	97.6	97.7
Cooling Rate (°C/min)	~6			
Recovery Temperature (°C)	~48.65			

## Data Availability

The data presented in this study are available on request from the corresponding author.
